# Emergence of Vibrio cincinnatiensis, a Rare Human Pathogen, in Urban Crows

**DOI:** 10.1128/spectrum.03925-22

**Published:** 2022-12-08

**Authors:** Eiji Soga, Kanae Sakaguchi, Shino Takizawa, Mizuki Tanabe, Tomohiro Denda, Shota Koide, Wataru Hayashi, Satoe Kasahara, Yukiko Nagano, Noriyuki Nagano

**Affiliations:** a Department of Health and Medical Sciences, Shinshu University Graduate School of Medicine, Nagano, Japan; b Department of Medical Sciences, Shinshu University Graduate School of Medicine, Science, and Technology, Nagano, Japan; c Suwa Hydrobiological Station, Faculty of Science, Shinshu University, Nagano, Japan; Institut National de Santé Publique du Québec

**Keywords:** human pathogen, urban crow, *Vibrio cincinnatiensis*

## LETTER

Currently, the genus *Vibrio* consists of 137 identified species (www.bacterio.net/vibrio.html [accessed 26 September 2022]); among them, Vibrio cincinnatiensis is one of the 12 *Vibrio* species that are considered potential human pathogens (1). To date, three cases of V. cincinnatiensis infections have been reported in humans (2–4). However, because of the diversity of disease presentation of V. cincinnatiensis, including septicemia and meningitis, enteritis, and skin and soft tissue infections in immunocompromised and immunocompetent elderly patients with no history of marine exposure, knowledge of the prevalence of this rare organism in environmental settings is essential (2–4).

**FIG 1:**
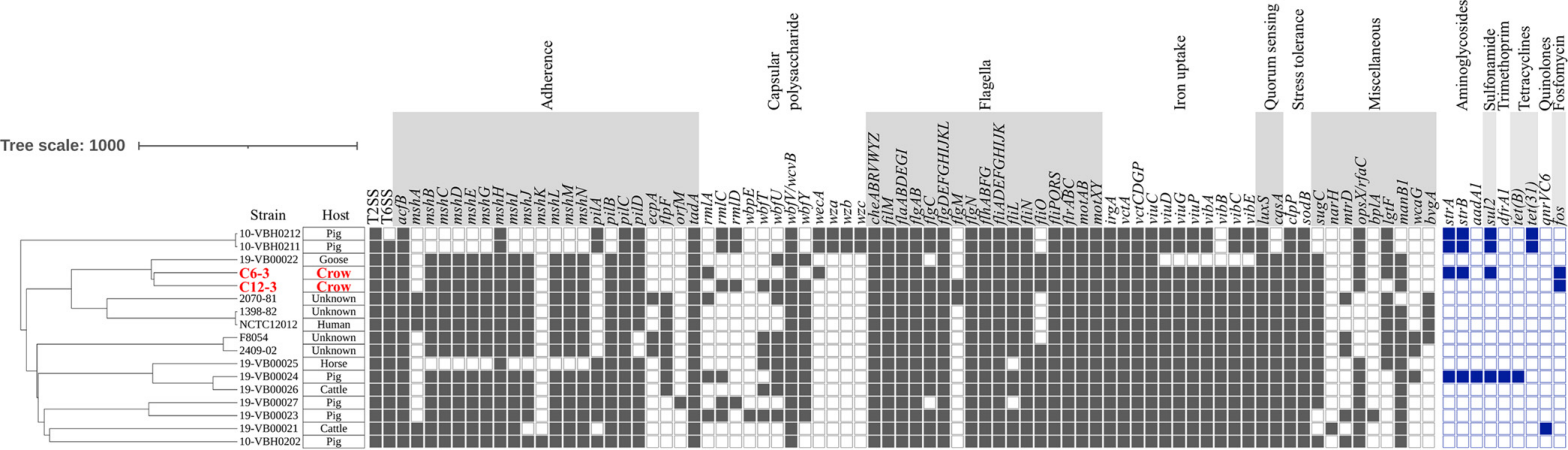
Whole-genome multilocus sequence typing-based phylogeny of 17 Vibrio cincinnatiensis strains with presence/absence matrices for virulence-associated and antimicrobial resistance genes. Filled boxes indicate the presence of genes. Strains C6-3 and C12-3 from this study are marked in red.

Here, V. cincinnatiensis strains C6-3 and C12-3 were detected using CHROMagar mSuperCARBA (Kanto Chemical Co., Tokyo, Japan). They were isolated from fresh droppings from two different crows, collected from below the roosting trees in a public park of Suwa City in October 2021. The geographic location of the sampling site was more than 110 km from the sea; therefore, the crows, which commonly migrate 10 to 20 km outward from their roosts to forage, were not likely to be associated with seawater exposure. The V. cincinnatiensis strains C6-3 and C12-3 grew on thiosulfate-citrate-bile salts-sucrose agar, exhibiting yellow colonies indicative of sucrose utilization. They were comma-shaped Gram-negative rods that were halophilic, oxidase positive, and motile. Matrix-assisted laser desorption ionization–time of flight mass spectrometry-based identification (BioTyper v3.1; Bruker Daltonics, Bremen, German) yielded low confidence values for V. cincinnatiensis (scores of 1.74 and 1.79 for C6-3 and C12-3, respectively). The assigned species were verified on the basis of biochemical properties, such as positivity for acid production from arabinose, inositol, salicin, and sucrose determined by using the Vitek 2 Compact system (v07.01 software; bioMérieux, Tokyo, Japan) with Vitek 2 GN ID cards and API 50 CH kits (bioMérieux). Whole-genome sequencing was performed using the DNBSEQ-G400 platform (MGI Tech, Shenzhen, China). Sequence reads were assembled *de novo* using SPAdes v3.12.0 (https://cab.spbu.ru/software/spades). Strains C6-3 and C12-3 had genome average nucleotide identity values of 99.35% and 99.40% (JSpeciesWS [http://jspecies.ribohost.com/jspeciesws]) and digital DNA-DNA hybridization values of 93.7% and 94.4% (TYGS [https://tygs.dsmz.de]), respectively, compared with the V. cincinnatiensis type strain NCTC12012 (GenBank assembly accession number GCA_900460255.1), and were confirmed as V. cincinnatiensis strains. A whole-genome multilocus sequence typing tree constructed using PGAdb-builder (http://wgmlstdb.imst.nsysu.edu.tw), with the addition of the genomes of 15 V. cincinnatiensis strains obtained from the NCBI database (see Table S2 in the supplemental material), classified the strains broadly into three clades, with strains C6-3 and C12-3 clustering together with human and animal strains (Fig. 1). A virulence gene analysis was performed using VFanalyzer with the Virulence Factor Database (VFDB) (http://www.mgc.ac.cn/VFs). The crow strains harbored virulence-associated gene repertoires, such as those encoding type II secretion system (T2SS), type VI secretion system (T6SS), flagellar, adherence, stress tolerance, and quorum-sensing proteins, which were comparable to those of other strains of human and animal origin (Fig. 1).

Antimicrobial resistance genes found with ResFinder v4.1 (https://cge.food.dtu.dk/services/ResFinder) and CARD (https://card.mcmaster.ca) are shown in Fig. 1. Antimicrobial susceptibility testing with the broth microdilution method, performed according to Clinical and Laboratory Standards Institute document M45 (5), revealed that the primary strain C6-3, which was negative for β-lactamase genes, was resistant to β-lactams, including carbapenems and trimethoprim-sulfamethoxazole, but was susceptible to gentamicin, amikacin, levofloxacin, and minocycline (see Table S1). The primary strain C12-3 exhibited resistance only to trimethoprim-sulfamethoxazole. These strains were found to have β-lactam and trimethoprim-sulfamethoxazole MIC values mainly below the susceptibility breakpoints after storage of the glycerol stock cultures at −80°C for 10 months (data not shown). Notably, both strains carried *fos* genes, with fosfomycin MIC values of >128 μg/mL, which remained unchanged between primary and stock strains (Fig. 1; also see Table S1).

The main limitation of this study was the small sample size from a limited geographic area. V. cincinnatiensis is rare, accounting for only 0.3% of the *Vibrio* species detected in aquatic environmental samples (6). Further studies are required to better estimate the geographic differences in the prevalence of this organism among crow individuals. Nonetheless, our work may shed light on the new role of urban crows as potential reservoirs and vectors for transmitting a rare human pathogen, V. cincinnatiensis, to humans and animals via a contaminated environment.

### Data availability.

Genome assemblies for strains C6-3 and C12-3 have been deposited in the DDBJ/EMBL/GenBank database under accession numbers GCA_022760875.1 and GCA_022760885.1, respectively.

## References

[1] Farmer JJ, III, Janda JM, Brenner FW, Cameron DN, Birkhead KM. 2015. *Vibrio*. *In* Trujillo ME, Dedysh S, DeVos P, Hedlund B, Kämpfer P, Rainey FA, Whitman WB (ed), Bergey's manual of systematics of archaea and bacteria, 3rd ed. John Wiley and Sons, Inc., Hoboken, NJ. doi:10.1002/9781118960608.gbm01078.

[2] Brayton PR, Bode RB, Colwell RR, MacDonell MT, Hall HL, Grimes DJ, West PA, Bryant TN. 1986. *Vibrio cincinnatiensis* sp. nov., a new human pathogen. J Clin Microbiol 23:104–108. doi:10.1128/jcm.23.1.104-108.1986. 2422196 PMC268580

[3] Wuthe HH, Aleksić S, Hein W. 1993. Contribution to some phenotypical characteristics of *Vibrio cincinnatiensis*: studies in one strain of a diarrhoeic human patient and in two isolates from aborted bovine fetuses. Zentralbl Bakteriol 279:458–465. doi:10.1016/S0934-8840(11)80417-2. 8305803

[4] Kunitomo K, Uemura N, Shimizu T, Hayano S, Tsuji T. 2022. Skin and soft tissue infections and bacteremia caused by *Vibrio cincinnatiensis*. IDCases 29:e01564. doi:10.1016/j.idcr.2022.e01564. 35845826 PMC9278064

[5] Clinical and Laboratory Standards Institute. 2016. Methods for antimicrobial dilution and disk susceptibility testing of infrequently isolated or fastidious bacteria, 3rd ed. CLSI document M45. Clinical and Laboratory Standards Institute, Wayne, PA. 10.1086/510431 17173232

[6] Kokashvili T, Whitehouse CA, Tskhvediani A, Grim CJ, Elbakidze T, Mitaishvili N, Janelidze N, Jaiani E, Haley BJ, Lashkhi N, Huq A, Colwell RR, Tediashvili M. 2015. Occurrence and diversity of clinically important *Vibrio* species in the aquatic environment of Georgia. Front Public Health 3:232. doi:10.3389/fpubh.2015.00232. 26528464 PMC4603242

