# Supplementary material for: Emergence of Vibrio cincinnatiensis, a Rare Human Pathogen, in Urban Crows
Source: Microbiol Spectr. 2022 Dec 8;11(1):e03925-22. doi: 10.1128/spectrum.03925-22 (PMC9927279; doi:10.1128/spectrum.03925-22)
Supplement: Supplemental file 1 — Tables S1 and S2. Download spectrum.03925-22-s0001.pdf, PDF file, 0.1 MB [file spectrum.03925-22-s0001.pdf]

TABLE S1 MICs of antimicrobial agents and interpretive categories of V. cincinnatiensis strains C6-3 and C12-3 from crow fecal samples.

Antimicrobial agents	Strain C6-3		Strain C12-3	
	MIC (μg/mL)	Interpretive category[a]	MIC (μg/mL)	Interpretive category[a]
Ampicillin	>16	R	4	S
Piperacillin	>64	R	64	I
Ampicillin-sulbactam	>16–8	R	4–2	S
Piperacillin-tazobactam	>64–4	R	64–4	I
Cefazolin	>16	R	4	I
Cefotiam	>4		1	
Ceftazidime	>16	R	2	S
Cefpodoxime	>4		≤1	
Ceftriaxone	>32		≤1	
Cefepime	>16	R	8	I
Cefmetazole	>32		8	
Flomoxef	>16		4	
Aztreonam	>16		16	
Imipenem	>8	R	2	I
Meropenem	8	R	0.25	S
Gentamicin	≤2	S	4	S
Amikacin	≤8	S	32	I
Levofloxacin	0.25	S	≤0.12	S
Fosfomycin	>128		>128	
Minocycline	≤2	S	≤2	S
Trimethoprim-sulfamethoxazole	>2–38	R	>2–38	R

[a]Interpretive categories from Clinical and Laboratory Standards Institute documents M45 was used.

TABLE S2 List of 15 *Vibrio cincinnatiensis* genomes

Strain	Country	Host species	Source	NCBI accession number
C6-3 (this study)	Japan	Crow	Feces	GCA_022760875.1
C12-3 (this study)	Japan	Crow	Feces	GCA_022760885.1
10-VBH0202	Germany	Pig	Fetus, amnion	GCA_022114085.1
10-VBH0211	Germany	Pig	Fetus, amnion	GCA_022114045.1
10-VBH0212	Germany	Pig	Unknown	GCA_022114065.1
19-VB00021	Germany	Cattle	Fetus abomasum	GCA_022113885.1
19-VB00022	Germany	Goose	Heart, brain	GCA_022113905.1
19-VB00023	Germany	Pig	Fetus, amnion	GCA_022113945.1
19-VB00024	Germany	Pig	Amnion	GCA_022113955.1
19-VB00025	Germany	Horse	Fetus, amnion	GCA_022113985.1
19-VB00026	Germany	Cattle	Abomasum	GCA_022114005.1
19-VB00027	Germany	Pig	Amnion	GCA_022114025.1
F8054	United States	Unknown	Unknown	GCA_009763885.1
1398-82	United States	Unknown	Cerebrospinal fluid	GCA_009763485.1
2070-81	United States	Unknown	Unknown	GCA_009763705.1
2409-02	United States	Unknown	Unknown	GCA_009665395.1
NCTC12012	United States	Human	Blood or cerebrospinal fluid	GCA_900460255.1
